# Inactivation of Digestive Proteases by Deconjugated Bilirubin and the Physiological Significance of Fasting Hyperbilirubinemia

**DOI:** 10.4021/gr2009.02.1270

**Published:** 2009-01-20

**Authors:** Xiaofa Qin

**Affiliations:** Department of Surgery, UMDNJ-New Jersey Medical School, 185 South Orange Avenue, Newark, NJ 07103, USA. Email: qinxi@umdnj.edu

**Keywords:** Hyperbilirubinemia, Fasting, Digestive proteases

## Abstract

It has been observed more than a century ago that in humans as well as in many animals, there was a significantly increase in blood bilirubin level during fasting. However, the physiological significance for this increase remains largely unknown. As it is found that digestive proteases are inactivated by free (or deconjugated) bilirubin, here I suggested that fasting hyperbilirubinemia would be a mechanism to save bilirubin during fasting to meet the anticipated increased needs to protect the gut against the damage by the increased luminal pancreatic proteases during and after feeding.

## To the editor

Fasting hyperbilirubinemia refers to the phenomenon of the remarkable increase of plasma bilirubin after food deprivation, which has been noted more than a century ago [1] and has been observed in humans [1], as well as in animals like monkeys [2], horses [3], and rats [4]. However, the physiological significance of fasting hyperbilirubinemia remains elusive. The finding of the role of bilirubin in the inactivation of digestive proteases would probably provide an explanation. Digestive proteases like trypsin and chymotrypsin are inactivated by free (or deconjugated) bilirubin but not conjugated bilirubin or biliverdin, and thus the inactivation of digestive proteases seems likely the evolutionary driving force for bilirubin or biliverdin predominance in animals [5]. Bilirubin would have played a critical role in the effective protection of the mucosa against the digestive damage by pancreatic proteases and the eventual inactivation of these enzymes. Large amounts of digestive proteases are produced and released during feeding, which would be accompanied by strikingly increased demand for bilirubin. Thus, the accumulation of bilirubin during fasting, as demonstrated by fasting hyperbilirubinemia, would be just a mechanism to save bilirubin during fasting to meet the anticipated increased needs to protect the gut against the damage by the increased luminal pancreatic proteases during and after feeding.
